# Physicians’ acceptance of pharmacists’ interventions in daily hospital practice

**DOI:** 10.1007/s11096-020-00970-0

**Published:** 2020-02-05

**Authors:** Rianne J. Zaal, Edwin W. den Haak, Elrozy R. Andrinopoulou, Teun van Gelder, Arnold G. Vulto, Patricia M. L. A. van den Bemt

**Affiliations:** 1 Department of Hospital Pharmacy, Erasmus Medical Center, University Medical Center Rotterdam, P.O. Box 2040, 3000 CA Rotterdam, The Netherlands; 2 Utrecht University, Utrecht Institute for Pharmaceutical Sciences, Utrecht, The Netherlands; 3 Department of Biostatistics, Erasmus Medical Center, University Medical Center Rotterdam, Rotterdam, The Netherlands; 4 Department of Internal Medicine, Erasmus Medical Center, University Medical Center Rotterdam, Rotterdam, The Netherlands; 5 Present Address: Department of Clinical Pharmacy and Pharmacology, University of Groningen, University Medical Center Groningen, Groningen, The Netherlands

**Keywords:** Acceptance rate, Clinical pharmacy, Drug-related problems, Interventions, Medication therapy management, Netherlands, Pharmaceutical care

## Abstract

*Background* The physicians’ acceptance rate of pharmacists’ interventions to improve pharmacotherapy can vary depending on the setting. The acceptance rate of interventions proposed by pharmacists located in the hospital pharmacy over the telephone and factors associated with acceptance are largely unknown. *Objective* To determine the physicians’ acceptance rate of pharmacists’ interventions proposed over the telephone in daily hospital practice and to identify factors associated with acceptance. *Setting* A retrospective case–control study was performed concerning adult patients admitted to a university hospital in the Netherlands. *Method* Pharmacists’ interventions, based on alerts for drug–drug interactions and drug dosing in patients with renal impairment, recorded between January 2012 and June 2013 that were communicated over the telephone were included. Factors associated with physicians’ acceptance were identified with the use of a mixed-effects logistic model. *Main outcome measure* The primary outcome was the proportion of accepted interventions. *Results* A total of 841 interventions were included. Physicians accepted 599 interventions, resulting in an acceptance rate of 71.2%. The mixed-effects logistic model showed that acceptance was significantly associated with the number of prescribed drugs (16 to ≤ 20 drugs OR_adj_ 1.88; 95% CI 1.05–3.35, > 20 drugs OR_adj_ 2.90; 95% CI 1.41–5.96, compared to ≤ 10 drugs) and the severity of the drug-related problem (problem without potential harm OR_adj_ 6.36; 95% CI 1.89–21.38; problem with potential harm OR 6.78; 95% CI 2.09–21.99, compared to clinically irrelevant problems), and inversely associated with continuation of pre-admission treatment (OR_adj_ 0.55; 95% CI 0.35–0.87). *Conclusion* Over the study period, the majority of pharmacists’ interventions proposed over the telephone were accepted by physicians. The probability for acceptance increased for patients with an increasing number of medication orders, for clinically relevant problems and for problems related to treatment initiated during admission.

## Impact on practice

- The majority of pharmacists’ interventions to improve pharmacotherapy in a Dutch hospital are accepted by physicians.
- Interventions regarding pre-admission treatment are less likely to be accepted.
- The patient’s general practitioner should be included in the discussion about recommendations regarding chronic pharmacotherapy.
- Further insight into physicians’ reasons for non-acceptance is necessary to optimize central pharmacy services.

## Introduction

To prevent drug-related problems, clinical pharmacists review medication orders from prescribers with the use of a clinical decision support system (CDSS). Recommendations to optimize pharmacotherapy may then be proposed to the prescriber. The acceptance rate of these interventions has been shown to vary between 52 and 100% [1–20]. This variation can be explained by differences in, among other things, the prescribing process (computerized or handwritten), the identification of potential drug-related problems (using CDSS or medication review), the medical ward (medical, surgical or intensive care unit) and the way of communicating the intervention (over the telephone, during ward rounds and/or electronically). Most previous studies on the physicians’ acceptance rate dealt with interventions proposed during ward rounds by clinical pharmacists who were a member of the multidisciplinary care team. However, in daily practice in the Netherlands and other West European countries a substantial number of interventions are proposed by pharmacists over the telephone. In most cases, these pharmacists are located in the hospital pharmacy and not at the medical ward.

A French multicentre study on pharmacists’ interventions published in 2015 considered a subset of interventions proposed by pharmacists from the hospital pharmacy over the telephone, and found that the acceptance rate was 62% [3]. The interventions had been extracted from a national database designed for documentation and classification of interventions during daily medication review. Since the number of interventions varied strongly between pharmacists, wards and hospitals, it is likely that not all interventions were documented.

Currently, the acceptance rate of pharmacists’ interventions communicated over the telephone is not exactly known. Besides, factors associated with the acceptance of pharmacist’s interventions proposed during daily routine over the telephone are little known.

Insight into the potential factors associated with acceptance could help optimize pharmacy services aimed at reducing drug-related problems and improving pharmacotherapy.

## Aims of the study

The aims of this study were to determine the acceptance rate of pharmacists’ interventions proposed over the telephone in the Netherlands and to identify potential risk factors associated with acceptance.

## Ethics approval

Since this study did not affect patient integrity, Erasmus Medical Center’s Medical Ethics Review Board waived approval for this study (Reference number MEC-2013-205).

## Method

### Design and setting

**Fig. 1:**
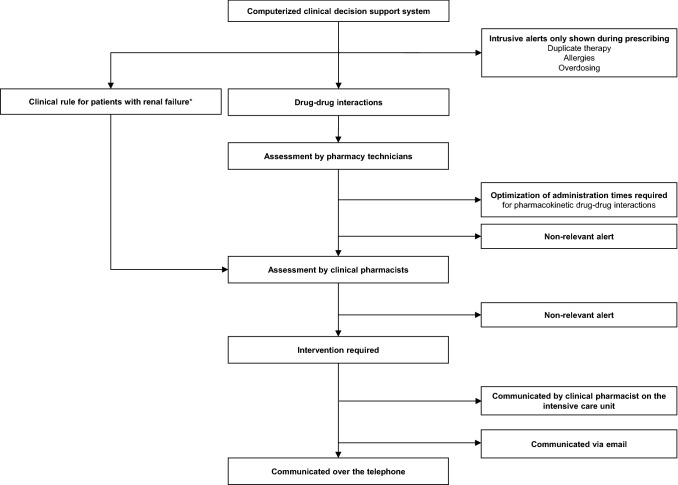
Schematic diagram of the clinical decision support system. *An electronic tool that combines medication data and laboratory values to detect patients with renal failure (glomerular filtration rate < 50 ml/min) at risk for drug-related problems

This study is a retrospective case–control study, performed in the central location of a university hospital in the Netherlands with 880 beds, including 62 beds for intensive care. In this hospital, medication is prescribed using a computerized physician order entry system (Medicator^®^, CSC-Isoft, Leiden, The Netherlands) combined with a clinical decision support system, based on the Dutch national drug database G-standard^®^ (Z-Index, The Hague, The Netherlands). This system generates intrusive alerts (pop-ups) relating overdosing, duplicate therapy, allergies and drug–drug interactions. Alerts that are not relevant in clinical practice are turned off. A schematic diagram of our CDSS and the handling of alerts is presented in Fig. 1.

A specific set of alerts is being handled by pharmacy technicians. They assess the clinical relevance of the alerts according to local standard operating procedures. Information on drug–drug interactions that probably require an intervention, other than optimizing administration times for pharmacokinetic drug–drug interactions which they discuss with the nurse, is forwarded to the pharmacists.

Reviewing alerts for drug–drug interactions is part of the daily routine of pharmacists in our hospital pharmacy. In addition, drug dosing for patients with renal impairment (glomerular filtration rate < 50 ml/min) is assessed with a clinical rule. This is an electronical tool that combines a patient’s characteristics, medication data and laboratory values to assess the risk for drug-related problems. Our rule is designed to identify contra-indicated drugs for patients with renal impairment or the necessity of dose reduction. All medication orders of patients identified by this clinical rule are reviewed by a pharmacist.

For a patient with drug-related problems deemed clinically relevant, the pharmacist in question provides a recommendation to the prescriber to optimize the pharmacotherapy—usually over the telephone. Interventions are recorded in the patient’s electronic medical record.

### Data collection

All pharmacists’ interventions recorded in the electronic medical records during weekdays from January 2012 until July 2013 resulting from drug–drug interactions and the clinical rule for patients with renal impairment were included in this study. Interventions not communicated over the telephone but by email were excluded from this study. Interventions for patients admitted to intensive care units were excluded as well, since a clinical pharmacist is present on these wards to handle alerts for these patients.

Data on the intervention (date, weekday, number of days since drug-related problem arose), the underlying drug-related problem [type of drug according to the Anatomical Therapeutic Chemical (ATC) classification system and whether the drug had been continued from pre-admission treatment or was initiated during admission], patient characteristics [age, gender, renal impairment (glomerular filtration rate < 50 ml/min), number of drugs prescribed at the time of intervention, length of stay at the time of intervention], pharmacist characteristics (gender and status: resident vs certified clinical pharmacist), and the prescriber’s medical specialty of were recorded. All patient data were processed anonymously in a protected database.

### Drug-related problems and interventions

Drug-related problems were classified according to the classification of Strand et al. [21], adapted by Leendertse et al. [22] The adapted classification differentiates drug-related problems by indication (additional drug therapy required or unnecessary drug therapy), effectiveness (ineffective drug therapy or subtherapeutic dosage), safety (adverse drug event or supratherapeutic dosage), drug use problems, and pharmaceutical care issues (monitoring, drug–drug interactions, contra-indicated drug, lifestyle, duplicate therapy). We added to this classification discrepancies between current treatment and pre-admission treatment as well as administrative prescribing errors (i.e. missing information on drug, dosage or administration route or duplicate orders).

The severity of drug-related problems was assessed using the National Coordinating Council for Medication Error Reporting and Prevention (NCC MERP) index, which classifies the severity ranging from clinically irrelevant problems that have the capacity to cause harm, to problems that may contribute to or result in death [23]. We grouped these categories into three classes, namely: clinically irrelevant drug-related problems (NCC MERP category A); drug-related problems without potential harm (NCC MERP category B–D); and drug-related problems with potential harm (NCC MERP E–I), varying from mild temporary discomfort to death. Because actual harm had been prevented by the intervention, we assessed the potential harm; i.e., possible consequences of the drug-related problem, in case the pharmacist should not have intervened. The assessment was performed separately by a hospital pharmacist/clinical pharmacologists (PvdB) and a physician/clinical pharmacologist (TvG). They discussed any discrepancies until consensus was reached.

Interventions were classified as proposed by Bedouch et al. [24] as drug choice (addition of a drug, discontinuation of a drug or drug switch), dose adjustment (increasing the dose, decreasing the dose), monitoring (subdivided into therapeutic drug monitoring, monitoring of biochemical parameters, recording an electrocardiogram and other types of monitoring) and optimization of administration times. Based on our experience, we added a class with other interventions, including consulting another specialist, reconciliation of pre-admission treatment, or administrative interventions.

### Outcomes

The primary outcome was the proportion of accepted interventions. The acceptance of interventions regarding drug choice, dose adjustments or optimization of administration was assessed by reviewing the computerized physician order entry system. Acceptance was defined as actual implementation of the suggested change in pharmacotherapy within 24 hours. We chose this time window because physicians do not always have the opportunity to change pharmacotherapy immediately and residents need to discuss some recommendations with their supervisors. Follow-up of recommendations regarding the monitoring of clinical chemical parameters or serum drug levels or performing an electrocardiogram was extracted from the medical records; interventions were considered as accepted when the suggested monitoring had been performed within 7 days. Monitoring of other adverse drug events—such as oedema, symptoms of heart failure or myalgia—could not be assessed in this study. Acceptance of these interventions was scored as unknown.

Characteristics of the intervention, the underlying drug-related problem, patient characteristics, pharmacist characteristics and the prescriber’s medical specialty were included as potential determinants for acceptance.

### Statistical analysis

The required sample size was calculated using the rule of thumb that at least 10 cases are required for every variable included in the analysis (sample size = 10*k/p, with k the number of variables and p the smallest proportion of negative and positive cases). Considering an expected acceptance rate of 60% and 15 potential predictors, the minimum required sample size was 375 interventions.

Descriptive analysis was performed using IBM SPPS Statistics version 21. A mixed-effects logistic model was performed with R statistical software version 3.2.2 (www.r-project.org) to investigate associations between potential determinants and acceptance, while accounting for multiple interventions within the same patients. The advantage of using mixed-effect models is that they can deal with unbalanced datasets, i.e. when the number of observations, which could be registered at different time points, per patient varies. This model was chosen because the acceptance of an intervention for a given patient could have been influenced by previous interventions for this patient. To ease the interpretation, the continuous variables age, number of drugs and length of stay were categorized into four categories, based on the quartiles of their frequencies. Adjusted odds ratios (OR_adj_), corrected for the other covariates, with 95% confidence intervals (95% CI) were calculated. A *p* value < 0.05 was considered statistically significant.

## Results

Data on 841 interventions, involving 623 patients, were included. Characteristics of these interventions and patients are presented in Table 1. Drug–drug interactions (46.4%), supratherapeutic dosages (21.8%) and requirement of additional drug therapy (8.7%) were the most common underlying drug-related problems (Table 2). Interventions were proposed most frequently for problems related to anti-infective agents (33.9%), drugs acting on blood and blood forming organs (13.4%), and drugs acting on the alimentary tract and metabolism (12.2%) (Table 3). As 599 of the 841 interventions had been accepted, the physicians’ acceptance rate was 71.2%.

**Table 1:** Characteristics of included patients and interventions included

	n	(%)
Patients	623	(100.0)
Female gender	263	(42.2)
Age in years, median (range)	64.0	[18–91]
DRPs^a^ per patient, median (range)	1.0	[1–6]
Proposed interventions	841	(100.0)

^*a*^*DRPs* drug-related problems

**Table 2:** Drug-related problems underlying pharmacists’ interventions (n = 841)

Class	Subclass of DRP	n	(%)
Indication	Additional drug therapy required	73	(8.7)
	Unnecessary drug therapy	3	(0.4)
Effectiveness	Ineffective drug therapy	8	(1.0)
	Dosage too low	45	(5.4)
Safety	Dosage too high	183	(21.8)
	Adverse drug event	8	(1.0)
Drug use	Drug use problem	13	(1.5)
Pharmaceutical care	Drug-drug interaction	390	(46.4)
	Contra-indication	64	(7.6)
	Duplicate therapy	25	(3.0)
	Monitoring	11	(1.3)
	Discrepancy with pre-admission pharmacotherapy	8	(1.0)
	Administrative prescribing errors	10	(1.2)
	Total	841	(100.0)

*DRP* drug-related problem

**Table 3:** Pharmacotherapeutic drug group underlying pharmacists’ interventions (n = 841)

Pharmacotherapeutic group^a^	n	(%)
Alimentary tract and metabolism (A)	103	(12.2)
Blood and blood forming organs (B)	113	(13.4)
Cardiovascular system (C)	69	(8.2)
Anti-infective agents for systemic use (J)	285	(33.9)
Antineoplastic and immunomodulating agents (L)	33	(3.9)
Musculo-skeletal system (M)	94	(11.2)
Nervous system (N)	86	(10.2)
Other^b^	58	(6.9)
Total	841	(100.0)

^a^According to Anatomical Therapeutic Chemical (ATC) classification system

^b^Includes dermatologicals (D), genito-urinary system and sex hormones (G), systemic hormonal preparations (H), antiparasitic products (P) and respiratory system (R)

The two clinical pharmacologists assessed 569 (67.7%) drug-related problems as clinically relevant problems with the potential to cause harm to the patient, whereas 253 (30.1%) of the problems were assessed as unlikely to cause harm. Nineteen (2.3%) problems were considered as clinically irrelevant.

The mixed-effects logistic model used to explore associations between potential risk factors and acceptance is presented in Table 4. Physicians’ acceptance was statistically significantly associated with the number of prescribed drugs (16 to ≤ 20 drugs OR_adj_ 1.88; 95% CI 1.05–3.35, > 20 drugs OR_adj_ 2.90; 95% CI 1.41–5.96) and with the severity of the drug-related problem (drug-related problems without potential harm OR_adj_ 6.36; 95% CI 1.89–21.38; drug-related with potential harm OR_adj_ 6.78; 95% CI 2.09–21.99), and inversely associated with continuation of pre-admission treatment (OR_adj_ 0.55; 95% CI 0.35–0.87).

**Table 4:** Association between potential risk factors and acceptance of pharmacists’ interventions (n = 769)^a^

Potential risk factor	OR_adj_	95% CI	*p*-value
**Characteristics of intervention**			
*Sequential count of interventions per patient*	1.00	0.77–1.31	0.976
*Type of intervention*			
Addition of a drug	Ref.		
Discontinuation of a drug	1.17	0.45–3.03	0.745
Drug switch	0.57	0.25–1.27	0.168
Increasing the dose	0.75	0.26–2.16	0.588
Decreasing the dose	0.81	0.33–2.01	0.653
Therapeutic drug Monitoring	0.47	0.16–1.41	0.179
Monitoring of biochemical parameters	0.53	0.19–1.45	0.219
Recording an electrocardiogram	0.33	0.10–1.08	0.067
Optimization of administration times	1.06	0.33–3.57	0.930
*Weekday of intervention*			
Monday	Ref.		
Tuesday	2.34	0.88–6.20	0.087
Wednesday	1.24	0.69–2.22	0.473
Thursday	0.83	0.45–1.50	0.532
Friday	0.94	0.51–1.74	0.852
*Number of days since problem arose*	0.93	0.76–1.14	0.477
**Characteristics of underlying drug-related problem**^**b**^			
*Pharmacotherapeutic group of drug involved*			
Alimentary tract and metabolism (A)	Ref		
Blood and blood system (B)	0.72	0.33–1.60	0.423
Cardiovascular system (C)	1.05	0.44–2.49	0.912
Anti-infectives for systemic use (J)	0.84	0.41–1.69	0.628
Antineoplastics and immunomodulating agents (L)	0.43	0.15–1.26	0.123
Musculo-skeletal system (M)	0.58	0.26–1.30	0.185
Nervous system (N)	0.65	0.27–1.52	0.320
Other^c^	0.57	0.23–1.41	0.220
*Severity*			
Clinically irrelevant drug-related problem	Ref.		
Relevant problem without potential harm	**6**.**36**	**1.89**–**21.38**	**0**.**002**
Relevant problem with potential harm	**6**.**78**	**2.09**–**21.99**	**0**.**001**
*Continuation of pre-admission treatment (hospital-initiated treatment is reference)*	**0**.**55**	**0.35**–**0.87**	**0**.**010**
**Patient characteristics**			
*Female gender*	0.86	0.58–1.27	0.443
*Age (years)*			
≤ 50	0.66	0.38–1.13	0.126
51 to ≤ 65	0.91	0.50–1.65	0.759
66 to ≤ 75	0.92	0.47–1.79	0.796
> 75			
*Presence of renal impairment*	1.10	0.70–1.73	0.676
*Number of drugs*			
≤ 10	Ref.		
11 to ≤ 15	1.63	0.94–2.81	0.082
16 to ≤ 20	**1**.**88**	**1.05**–**3.35**	**0**.**033**
> 20	**2**.**90**	**1.41**–**5.96**	**0**.**004**
*Length of stay (days)*			
≤ 1	ref.		
2 to ≤ 3	0.65	0.38–1.12	0.123
4 to ≤ 8	0.98	0.54–1.76	0.936
> 8	0.80	0.44–1.46	0.461
**Pharmacists’ characteristics**			
*Female gender*	1.53	0.89–2.60	0.122
*Residents (certified hospital pharmacists are reference)*	0.95	0.62–1.45	0.804
*Prescriber’s medical specialty*			
Medical	Ref.		
Surgical	1.14	0.71–1.84	0.589
Cardiology	1.09	0.65–1.82	0.754

Figures in bold are statistically significant

*OR* odds ratio, *CI* confidence interval, *Ref.* reference, *DRP* drug-related problem

^a^Excluding interventions for which acceptance could not be assessed (n = 51). Subsequently, interventions with missing data [renal function (n = 11) and continuation of pre-admission treatment (n = 6)] and interventions for intensive care units (n = 1) and intervention types “consulting another specialist” (n = 2) or “administrative interventions” (n = 1) were excluded from the mixed-effects model as well

^b^According to Anatomical Therapeutic Chemical (ATC) classification system

^c^Includes dermal preparations (D), genito-urinary system and sex hormones (G), systemic hormonal preparations (H), antiparasitic products (P) and respiratory system (R)

## Discussion

In this study, the physicians’ acceptance rate of pharmacists’ interventions was 71.2%. Acceptance was statistically significantly associated with the number of medication orders at the time of the intervention, the continuation of pre-admission treatment and the severity of the underlying drug-related problem.

This acceptance rate is somewhat higher than the acceptance rate of 62% found in the French multi-centre study referred to in the introduction section [3]. Others have reported even lower acceptance rates of around 50% [6, 12, 13]. The higher acceptance rate found in our study could perhaps be explained by the accurate assessment of the clinical relevance of potential drug-related problems by our pharmacists, illustrated by the small number of clinically irrelevant alerts.

In addition, differences such as the communication methods, the system for detecting the drug-related problems and physicians’ attitude towards pharmacists between the studies could have contributed to the discrepant acceptance rates.

The acceptance rate in our study is comparable with some reported acceptance rates of around 60% to 80% in settings where pharmacists have been integrated in the medical team on the ward [3, 13, 17, 18, 25]. Therefore, central checking of CDSS alerts and clinical rules might be commendable, also for settings with pharmacists on the ward, so that these pharmacist can focus on other drug-related problems, such as adverse drug reactions.

In our study, the number of prescribed drugs of a patient at the time of the intervention was significantly associated with acceptance. This may be related to the possibility of physicians having insufficient overview of a patient’s pharmacotherapy with a higher number of medication orders—and then are more inclined to accept a pharmacist’s intervention. This suggests an added value of the input of pharmacists for patients with polypharmacy, which is a well-known risk factor for drug-related problems [26].

Furthermore, the probability for acceptance decreased if the underlying drug had already been initiated before admission, which suggests that physicians may be reluctant to change the medication, initiated by another physician before admission, on which a patient has been stable on.

Our finding that interventions for drug-related problems assessed as clinically relevant are more likely to be accepted suggests that physicians carefully consider the clinical relevance of a problem—weighing the risks and benefits for the individual patient. On the other hand, their tendency not to accept clinically irrelevant interventions was in line with the clinical pharmacologist’s assessment.

We did not find an association between acceptance and any other characteristics of the intervention, the underlying problem, the patient or the prescriber. In addition, we found no difference in acceptance rates between pharmacy residents and certified hospital pharmacists, indicating that our residents are well trained to review pharmacotherapy and propose interventions. It may well be, however, that the physicians are not aware of the professional status of the pharmacist who proposed the intervention. Still, the finding that some of the drug-related problems were assessed as clinically irrelevant by clinical pharmacologists shows variability between different professionals with regard to the medication review process, despite training and use of guidelines.

In contrast to our results, Bedouch et al. [3] showed a significant association between several therapeutic drug classes and acceptance. Besides, some previous studies have shown differences in acceptance between surgical and medical wards [3, 8, 27]. We were not able to reproduce the results of these studies, which can probably be explained by differences in setting and the smaller sample size of our study.

In our setting, interventions were discussed between the pharmacist and the physician over the telephone and recorded in the patient’s electronic medical record. In our study, the pharmacy tab in the electronic medical records of the 623 included patients was viewed 5568 times in total; in 96.9% of cases by a pharmacist and in only 3.1% of cased by a physician. This indicates that physicians’ decisions to accept interventions generally are based on the discussion with the pharmacist, since they hardly viewed the recorded recommendations. Previous studies indeed have shown that verbally communicated interventions are much more likely to be accepted than interventions that are only recorded electronically [6, 25]. These findings support the feasibility and safety of pharmacists located in the hospital pharmacy proposing interventions for drug-related problems over the telephone.

Several limitations of our study need to be addressed. First, physician’s reasons for not accepting, that could have been discussed during the phonecall, had not been recorded systematically in the electronic records. Therefore, we could not assess whether there was a valid argument for not accepting the intervention in this study. In a future prospective study, it may be commendable to record physicians’ argumentations.

Second, the proportion of the proposed interventions in relation to the total number of CDSS alerts is unknown. In a previous study only 1.6% of all alerts required a pharmacist intervention [13]. This proportion could vary between different pharmacists and settings and could influence the acceptance rate. For example, the acceptance rate would perhaps be higher if the pharmacists decide to propose only the most urgent interventions. Given the small number of clinically irrelevant problems in our study, it is likely that all pharmacists involved focused on the most relevant problems and that differences between pharmacists had no significant effect on the acceptance rate.

Third, this study was performed in a single center. The findings may be difficult to extrapolate to other hospitals, especially those where the pharmacists have been more integrated in the ward medical. Fourth, we were not able to include any characteristics of the prescribers, except for specialty. In a previous study, physician’s status (resident vs specialist) has been associated with acceptance [25]. Besides, we could not determine whether an intervention was proposed to the initial prescriber or to another physician. It is not unthinkable that the acceptance rate can be influenced by proposing an intervention to the initial prescriber or to another physician.

Despite these limitations, the findings of this study may be of value to hospitals with central pharmacy services, where pharmacists are not integrated in the medical teams on the ward, to optimize their services and reduce drug-related problems.

Prospective follow-up of interventions and exploring physicians’ reasons for non-acceptance are recommended for future research, with the ultimate goal to minimize interventions that are irrelevant given the patient’s current medical condition. Furthermore, clinical consequences of non-acceptance in terms of patient harm, length of stay and pharmaceutical costs need to be studied.

To improve clinical pharmacy services, pharmacists and physicians in primary and secondary care should agree on their responsibilities on chronic pharmacotherapy during a patient’s admission, as we found that physicians tend to decline interventions regarding medication initiated before admission. Non-urgent recommendations could be discussed together with a patient’s general practitioner. Stronger arguments should be used to increase the acceptance of interventions for patients using fewer than 10 prescribed drugs. On the other hand, pharmacists could more pro-actively review the pharmacotherapy of patients using more than 15 prescribed drugs, to detect additional drug-related problems and optimize therapy together with the physician.

## Conclusion

In conclusion, during the period under study, most of the pharmacists’ interventions communicated over the telephone were accepted by the physicians. The probability of acceptance increased for patients with an increasing number of medication orders, for patients with relevant drug-related problems and for patients whose drug treatment had been initiated during admission. To optimize pharmacy services, further insight into physicians’ reasons for non-acceptance should be obtained.
